# Waste-Activated Sludge Fermentation for Polyacrylamide Biodegradation Improved by Anaerobic Hydrolysis and Key Microorganisms Involved in Biological Polyacrylamide Removal

**DOI:** 10.1038/srep11675

**Published:** 2015-07-06

**Authors:** Xiaohu Dai, Fan Luo, Dong Zhang, Lingling Dai, Yinguang Chen, Bin Dong

**Affiliations:** 1 State Key Laboratory of Pollution Control and Resources Reuse, School of Environmental Science and Engineering, Tongji University, 1239 Siping Road, Shanghai 200092, China; 2 Guangzhou Municipal Engineering Design & Research Institute, 348 Huanshi East Road, Guangzhou, 510060.

## Abstract

During the anaerobic digestion of dewatered sludge, polyacrylamide (PAM), a chemical conditioner, can usually be consumed as a carbon and nitrogen source along with other organic matter (e.g., proteins and carbohydrates in the sludge). However, a significant accumulation of acrylamide monomers (AMs) was observed during the PAM biodegradation process. To improve the anaerobic hydrolysis of PAM, especially the amide hydrolysis process, and to avoid the generation of the intermediate product AM, a new strategy is reported herein that uses an initial pH of 9, 200 mg COD/L of PAM and a fermentation time of 17 d. First, response surface methodology (RSM) was applied to optimize PAM removal in the anaerobic digestion of the sludge. The biological hydrolysis of PAM reached 86.64% under the optimal conditions obtained from the RSM. Then, the mechanisms for the optimized parameters that significantly improved the biological hydrolysis of PAM were investigated by the synergistic effect of the main organic compounds in the sludge, the floc size distribution, and the enzymatic activities. Finally, semi-continuous-flow experiments for a microbial community study were investigated based on the determination of key microorganisms involved in the biological hydrolysis of PAM.

Polyacrylamide (PAM), a linear water-soluble polymeric compound with a high molecular weight, is widely used in wastewater treatment processes^1^,^2^, papermaking^3^, soil conditioning^4^,^5^, irrigation furrow anticorrosives^6^ and oil production^7^. Although PAM is generally accepted as a non-toxic substance^8^, the incompletely biodegraded intermediate products, especially the acrylamide monomer (AM), is hazardous to the peripheral nerves. The large production and utilization of PAM release a significant amount of this material into the environment and pose potential risks to humans and other organisms. The increasing use of PAM, mainly in industries, introduces it into wastewater treatment plants (WWTPs), which are the last barriers prior to PAM’s environmental release. In WWTPs, activated sludge technology is a widely used biological method of wastewater treatment; however, large amounts of waste activated sludge (WAS) are produced during this process. Fortunately, an anaerobic digestion technique was applied to reduce the sludge volume and produce the energy biogas via anaerobic microbes^9^,^10^,^11^. Additionally, the new high-solid anaerobic digestion process, which utilizes a smaller reactor volume and lower energy consumption^12^, may gradually replace the traditional low-solid anaerobic digestion processes. However, large amounts of chemical conditioners such as PAM (e.g., 2–5 gram per kilogram of the total solids present) are added to enhance the dehydration ratio of the sludge during this process. Therefore, PAM is present in the sludge and needs to be degraded as thoroughly as possible. One method that could be used to improve PAM biodegradation is to simultaneously biodegrade both the PAM and the sludge in an anaerobic fermentation system; this is possible because both PAM and sludge can be consumed as carbon sources^13^.

Recent studies concerning the biodegradation of PAM have mainly focused on the limited hydrolysis rate due to particle aggregation^14^,^15^. For example, PAM from soil or oilfield wastewater can be hydrolyzed as a nitrogen (i.e., amide hydrolysis) or carbon (i.e., carbon chain hydrolysis) source during an aerobic or anaerobic process. Similarly, Haveroen *et al.*^16^, El-Mamouni *et al.*^17^ and Nakamiya *et al.*^18^ reported that PAM from sewage sludge could be hydrolyzed as a carbon or nitrogen source during an anaerobic or aerobic process (Table S1, Supplementary Information). However, several studies revealed that PAM from sewage sludge or riverbed mud might be difficult to hydrolyze during an anaerobic or aerobic process^15^,^19^. These results indicated that different types of PAM from different substrates might show dissimilar biodegradation behaviors during an anaerobic or aerobic process; however, a significant accumulation of AM (e.g., approximately 500 milligram per kilogram of the total solids present) was observed during the PAM biodegradation process. As a consequence, a new strategy should be developed to improve the anaerobic hydrolysis of PAM, especially by accelerating the amide hydrolysis process, to avoid the generation of the intermediate product AM.

Because recent investigations into improving the biodegradation of PAM have become an interesting topic^20^,^21^,^22^,^23^,^24^, many researchers have begun to biodegrade PAM using isolated microorganisms in pure cultures or other co-metabolism substrates. Previous publications noted that *Bacillus* isolated from contaminated soil and oilfield activated sludge could biodegrade PAM to 36.3% of its original mass^20^, and sulfate-reducing *Bacteria* could improve the PAM biodegradation to 61.2% by using PAM as the only carbon source^21^. Additionally, fungus isolated from soil (i.e., white-rot fungus) could mineralize PAM after being solubilized by fungal peroxidases or cellobiose dehydrogenase with a PAM removal efficiency of 19%^22^,^23^. These results indicated that different types of microorganisms had different effects on PAM’s biodegradation; they also showed the biological inhibitory effects of PAM. However, to date, the strategy to enhance the biological hydrolysis of PAM by regulating key microorganisms involved in the biological hydrolysis of PAM removal in WAS fermentation systems has not been investigated.

To further improve the anaerobic hydrolysis of PAM (i.e., amide hydrolysis), a new strategy is proposed in this study that uses an initial pH of 9, 200 mg COD/L of PAM and a fermentation time of 17 d. The mechanisms for the optimized parameters that significantly improved the biological hydrolysis of PAM were investigated, and semi-continuous-flow experiments regarding the key enzyme activities as well as a microbial community study were performed.

## Results

### Optimized Biological Hydrolysis of PAM using the Response Surface Methodology

The response surface methodology (RSM) was applied to optimize PAM removal (i.e., amide hydrolysis) during the anaerobic digestion of sludge. To minimize the influence of uncontrolled variables on the RSM, five-level, three-variable central composite design (CCD) experiments with 20 runs were randomly conducted to optimize the initial pH (*X*_*1*_), PAM amount (*X*_*2*_) and fermentation time (*X*_*3*_) to improve the biological hydrolysis of PAM, as shown in Table 1. The batch experiments designed by CCD are illustrated using the values in Table 1, and both the maximal biological hydrolysis of PAM for the alkaline and acidic conditions are shown in Table 1. Using multiple regressions to analyze the data from Table 1, the following second-order polynomial equations for the biological hydrolysis of PAM (i.e., equation-1 and equation-2) were generated:
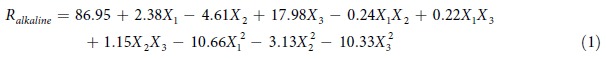

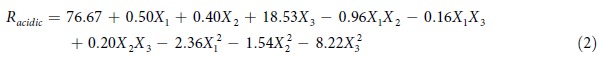
where *R*_*alkaline*_ is the predicted response of the biological hydrolysis of PAM under alkaline conditions; *R*_*acidic*_ is the predicted response of the biological hydrolysis of PAM under acidic conditions; and *X*_*1*_, *X*_*2*_ and *X*_*3*_ are the three independent factors described previously. As shown in Table S2 in the Supplementary Information, the statistical significance of the models and their terms were evaluated by an analysis of variance (ANOVA). These two models of *F* showed a low *P*-value (< 0.001), which implied the high significance of the quadratic model. The values of the predicted *R*^*2*^ and the adjusted *R*^*2*^ were sufficiently high to support the significance of the models. The precision, which is an indicator of the signal-to-noise ratio, must be greater than 4 and was found to be sufficiently large to verify the effectiveness of the application of these models. Concurrently, the relatively low coefficients of variation (CV = 9.33% for *R*_*alkaline*_ and CV = 11.49% for *R*_*acidic*_) indicated that these two models showed good precisions and reliabilities. Additionally, the predicted versus actual plots of the biological hydrolysis of PAM in the alkaline and acidic conditions were distributed relatively near to the straight line for both responses (Figure S1, Supplementary Information); the normal probability plots of the studentized residuals also exhibited an approximately linear pattern (Figure S2, Supplementary Information), which supported the reliability of the models.

### Synergistic Effect of the Main Organic Compounds in the Sludge on the Biological Hydrolysis of PAM

**Figure 1:**
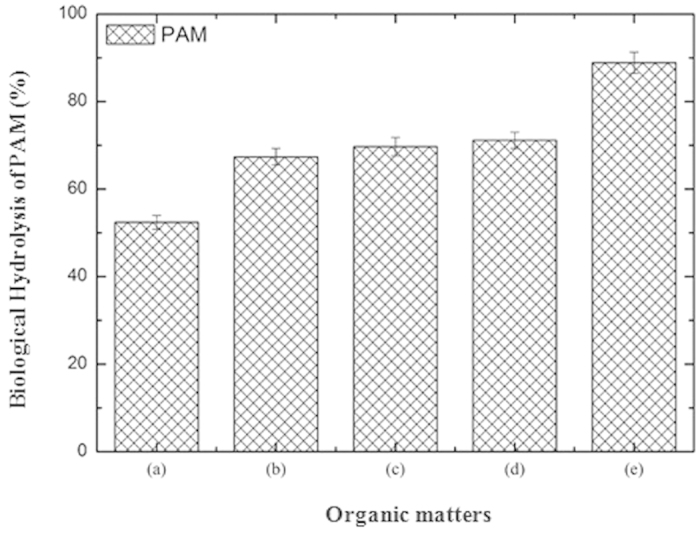
Effect of the main organic matter in the sludge and the initial pH on the biological hydrolysis of PAM in the synthetic wastewater batch experiments after 17 d. (**a**) PAM; (**b**) PAM + starch; (**c**) PAM + BSA; (**d**) PAM + starch + BSA; and (**e**) PAM + starch + BSA (pH 9.0). The error bars represent the standard deviations of triplicate tests.

Because the main organic compounds of the WAS include carbohydrates and proteins, the consumption of carbohydrates and proteins might affect the biological hydrolysis of PAM. Figure 1 illustrates the impact of these types of organic matter on the biological hydrolysis of PAM during the synthetic wastewater batch experiments with a fermentation time of 17 days. It was observed that the biological hydrolysis of PAM was 67.4 ± 1.9% and 69.7 ± 2.1%, respectively, when carbohydrates (i.e., starch as model) or proteins (i.e., BAS as model) were added into the PAM synthetic wastewater. However, the biological hydrolysis of PAM reached 88.9 ± 2.4% by co-fermentation with carbohydrates and proteins together at pH 9.0 for 17 d.

### Effect of the Fermentation pH on the Cumulative Floc Size Distribution with regard to the Enhancement of the Biological Hydrolysis of PAM

**Figure 2:**
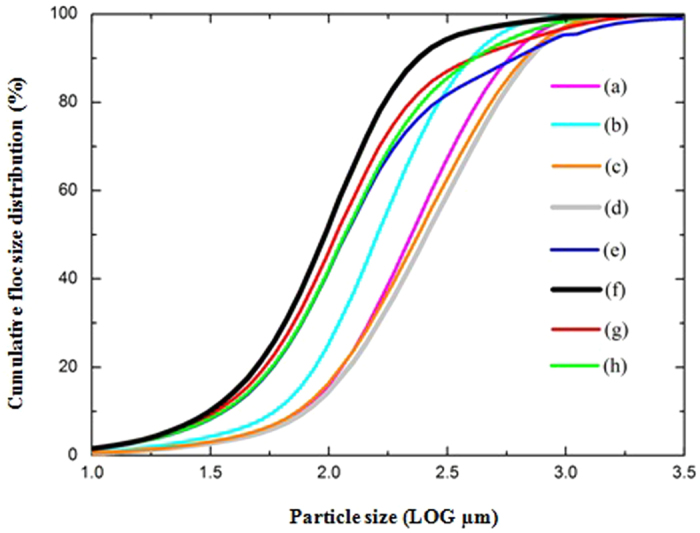
Effect of the fermentation pH on the sludge floc size distribution with a fermentation time of 17 day. (**a**) pH 4.0; (**b**) pH 5.0; (**c**) pH 6.0; (**d**) pH 7.0; (**e**) pH 8.0; (**f**) pH 9.0; (**g**) pH 10.0; and (**h**) pH 11.0.

Considering that the sludge floc size might affect the biological hydrolysis of PAM due to changes in the fermentation pH^25^,^26^,^27^, the cumulative floc size distribution of the sludge containing PAM at different pHs (e.g., from a pH of 4.0 to 11.0) was investigated, and the sludge floc median diameter was determined, as shown in Fig. 2 and Table S3 (Supplementary Information). In Fig. 2, which describes the influence of the fermentation pH on the sludge cumulative floc size distribution, the observed sludge floc size of 90% of the cumulative volume was approximately 178 μm (2.25 LOG μm = 178 μm) when the sludge was fermented at an initial pH of 9.0 for 17 d, whereas the sludge floc size was greater than 178 μm when the sludge was fermented at all other initial pHs. In Table S3, which shows the effect of fermentation pH on the sludge floc median diameter and the biological hydrolysis of PAM, the observed sludge floc median diameter was found to be approximately 109 μm, and the hydrolysis of PAM was determined to be 86.8 ± 3.4% after the sludge was fermented at an initial pH of 9.0. However, the sludge floc media diameter was more than 109 μm, and the hydrolysis of PAM was less than 86.8 ± 3.4% when the sludge was fermented at all other initial pHs.

### Determination of the Key Microorganisms Involved in the Biological Hydrolysis of PAM

**Figure 3:**
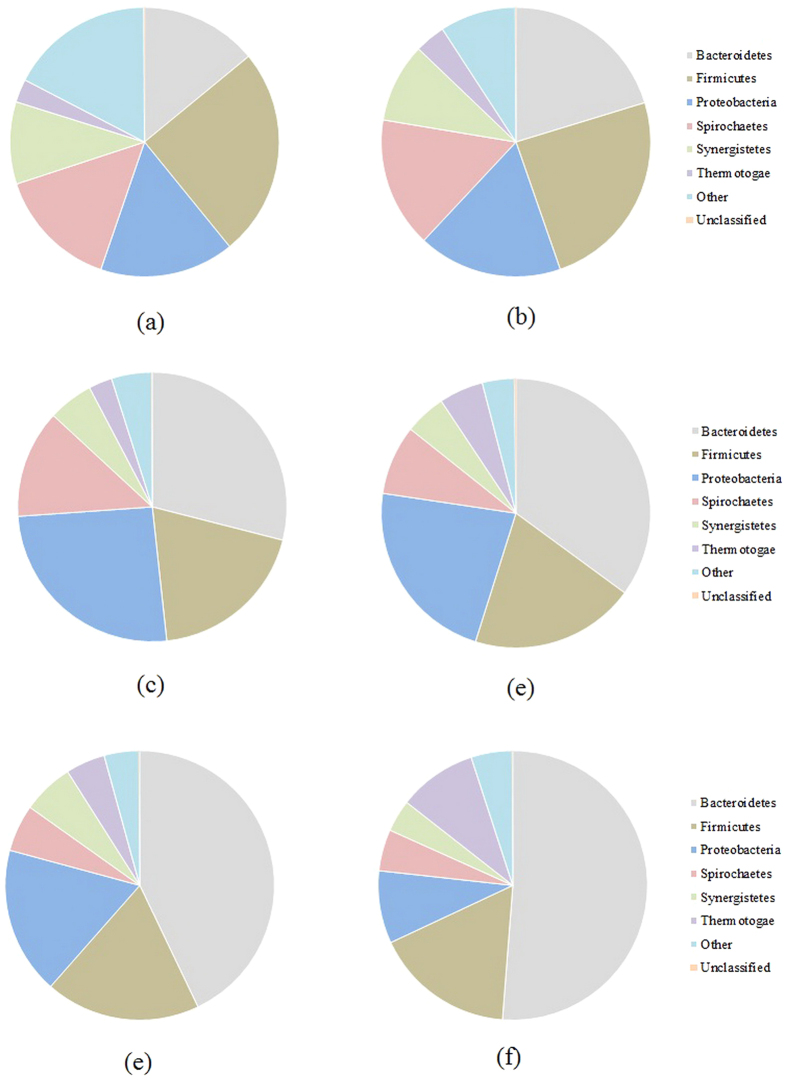
Phylum-level distributions of *Bacterial* populations involved in the biological hydrolysis of PAM with different fermentation times. (**a**) 1 d; (**b**) 4 d; (**c**) 7 d; (**d**) 14 d; (**e**) 19 d; and (**f**) 30 d.

Considering that the microbial distribution characteristics are important to determining the key microorganisms involved in the biological hydrolysis of PAM, a batch experiment with PAM-rich synthetic wastewater was conducted. The samples were collected from the anaerobic reactor after different fermentation times (e.g., 1, 4, 7, 14, 19 and 30 d) to determine the key microorganisms involved in the biological hydrolysis of PAM; the microbial community structures were studied by 454 pyrosequencing (Fig. 3). To estimate the phylogenetic diversities of the *Bacterial* communities in the anaerobic reactor after the different fermentation times listed above, the trimmed sequences were grouped into operational taxonomic units (OTUs) using a 97% identity threshold. Table S4 shows that the *Bacterial* sequences of the anaerobic reactor at the different fermentation times of 1, 4, 7, 14, 19 and 30 d were identified to contain 684, 764, 740, 762, 957, and 722 OTUs, respectively, and the sufficient coverage (e.g., more than 86%) found in each sample suggested that the 454 pyrosequencing method had captured the most frequently occurring microorganisms. Most *Bacteria* in the anaerobic reactor with different fermentation times were part of the phyla *Bacteroidete***s**, *Firmicutes*, *Proteobacteria*, and *Spirochaetes*. Additionally, the phyla *Bacteroidete*s, *Firmicutes*, *Proteobacteria*, and *Spirochaetes* were present after 1 day of fermentation and accounted for 14.04%, 25.09%, 16.12% and 14.70%, respectively, of all of the *Bacterial* sequences found; after 30 days of fermentation, these values were 51.23%, 16.80%, 8.71% and 4.98%, respectively. These results indicated that a longer running time increased the relative abundance of *Bacteroidete*s in the anaerobic reactor. Table S5 (Supplementary Information) shows the Pearson correlation analysis between the biological hydrolysis of PAM and the microbial community; among the six major phyla (i.e., *Bacteroidetes*, *Firmicutes*, *Proteobacteria*, *Spirochaetes*, *Synergistetes* and *Thermotogae*) generated from the anaerobic system, *Proteobacteria* was found to correlate significantly with the biological hydrolysis of PAM.

### Effect of an Alkaline pH on the Functional *Bacterial* and *Archaea* Populations Involved in the Biological Hydrolysis of PAM

**Figure 4:**
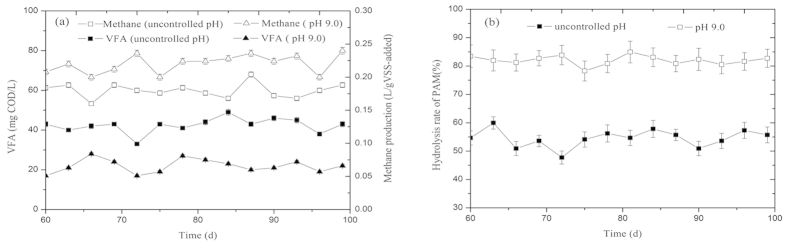
VFA accumulation, methane production (a), and the biological hydrolysis of PAM (b) in fermentation reactors with an initial pH of 9 or uncontrolled pH during a fermentation period of 100 d. The error bars represent the standard deviation of triplicate measurements.

Based on the optimized parameters mentioned above, two anaerobic reactors were operated with an additional 200 mg COD/L of PAM and a 17-day fermentation time at an initial pH of 9.0 and at an uncontrolled pH. The variations of VFA, methane and PAM in the fermentation reactors with an initial pH of 9.0 and with an uncontrolled pH during the fermentation period of 100 days are shown in Fig. 4. The average concentration of VFAs in the reactor with an initial pH of 9.0 was only 21.9 ± 3.4 mg/L, whereas that of the uncontrolled pH reactor was 42.4 ± 3.8 mg/L (Fig. 4a). The methane production of the fermentation reactor with an initial pH of 9.0 (0.18–0.22 L/g VSS-added) was found to be significantly higher than that of the reactor with an uncontrolled pH (0.15–0.20 L/g VSS-added). Additionally, the biological hydrolysis of PAM in the fermentation reactor with an initial pH of 9.0 was improved significantly (Fig. 4b). Thus, utilizing an initial fermentation pH of 9.0 significantly enhanced the biological hydrolysis of PAM, increased the methane production, and accelerated the utilization of VFA.

## Discussion

**Figure 5:**
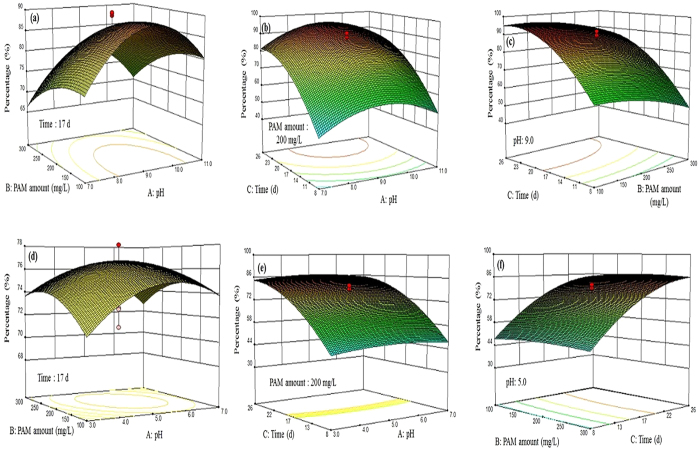
Three-dimensional surface and contour plots for the biological hydrolysis of PAM under alkaline (a–c) and acidic (d–f) conditions.

The three-dimensional response surfaces and the contour plots for the biological hydrolysis of PAM under alkaline and acidic conditions are shown in Fig. 5 by holding one factor to be constant at zero and varying the another two factors. The results in Fig. 5a–c show that an ineffective biological hydrolysis of PAM can occur in subsequent fermentation when the alkaline fermentation was not operated at the following conditions: pH of 7–9, 100–300 mg/L of PAM, and a fermentation time 8–26 d. Additionally, the results in Fig. 5d–f show that an ineffective biological hydrolysis of PAM can occur in subsequent fermentation when the acidic fermentation was not operated at the following conditions: pH of 3–7, 100–300 mg COD/L of PAM, and a fermentation time of 8–26 d. Thus, the maximum biological hydrolysis of PAM was expected to occur at the intersection of the zero levels of all three factors.

Figure 5a shows that the biological hydrolysis of PAM first increased when the amount of PAM changed from 100 to 200 mg/L and then decreased when the amount of PAM increased from 200 to 300 mg/L in the pH range from 7.0 to 11.0. Figure 5b shows that the fermentation time (e.g., from 8 to 17 d) led to an increase in the biological hydrolysis of PAM at a lower pH range (e.g., 7.0–9.0); however, a longer fermentation time (e.g., > 17 d) resulted in a slight decrease in the biological hydrolysis of PAM of a higher pH range (e.g., 9.0–11.0). Similarly, Fig. 5d showed that the increase in the amount of PAM (e.g., from 100 to 200 mg/L) increased the biological hydrolysis of PAM, and that the higher values of PAM (from 200 to 300 mg/L) decreased the biological hydrolysis of PAM in the pH range from 3.0 to 7.0. As shown in Fig. 5e, the biological hydrolysis of PAM increased as the fermentation time increased from 8 to 17 d at the lower pH range (e.g., 3.0–5.0) and exhibited a slight decrease when the fermentation time exceeded 17 d at the higher pH range (e.g., 5.0–7.0).

Figure S3 (Supplementary Information) shows the overlay plots for the biological hydrolysis of PAM (i.e., amide hydrolysis) by setting the amount of PAM and pH as variable factors. The plots were determined by setting the amount of PAM and the pH to be variable factors and setting the fermentation time equal to 17 d. By defining the desired biological hydrolysis of PAM in the alkaline and acidic conditions, the shaded portion of the overlay plots was considered to be the satisfactory zone (Figure S3, Supplementary Information). The optimal conditions (i.e., a pH of 9.0, 200 mg COD/L of PAM and a fermentation time of 17 d) were obtained from the overlay plots. Under such conditions, the predicted biological hydrolysis of PAM (i.e., amide hydrolysis) was 86.95 ± 4.5% and the corresponding experimental value was 86.64 ± 4.2%. Thus, the model predictions were found to be similar to the experimental data. Because the main purpose of this study was to enhance the biological hydrolysis of PAM, the reasons why the optimal conditions listed above produced an improved hydrolysis of PAM compared to the other methods were investigated.

Compared to that using only carbohydrates or proteins, the biological hydrolysis of PAM was improved significantly by co-fermentation with carbohydrates, proteins and PAM together, suggesting that there was a synergistic effect between the carbohydrates, proteins and PAM in the anaerobic fermentation process. According to our previous study, the activities of the sludge anaerobic fermentative enzymes and the key microorganisms could be enhanced significantly by adding carbohydrates to the sludge anaerobic fermentation system^28^; thus, an analogous synergistic effect could be seen in the anaerobic co-fermentation system including carbohydrates, proteins and PAM. Besides, it could be concluded that fermenting sludge at an initial pH of 9.0 caused significantly smaller floc sizes (Fig. 2 and Table S3), which resulted in an increased contact area between the PAM and the microorganisms, improving the biological hydrolysis of PAM. Thus, fermenting sludge at an initial pH of 9.0 reduced the sludge floc size and provided a suitable contact area between the PAM and the microorganisms, resulting in a significantly improved biological hydrolysis of PAM.

**Figure 6:**
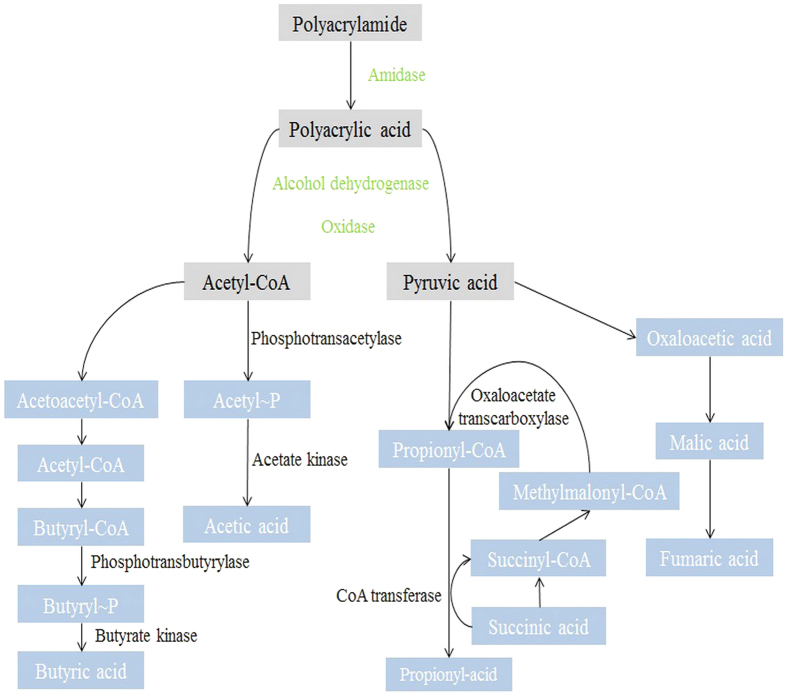
Proposed metabolic pathways for the biological hydrolysis of PAM. Only the key enzymes detected in this study are labeled.

The above observations could also be explained with respect to the enzymes. According to the proposed metabolic pathways for the biological hydrolysis of PAM (Fig. 6), PAM is first hydrolyzed into polyacrylic acid by AM and then into acetyl-CoA and pyruvic acid by ADH. In the metabolic pathway, butyryl-CoA and acetyl-CoA are, respectively, converted into butyryl and acetyl phosphate by PTB and PTA. Then, the butyryl and acetyl phosphate are converted into butyric and acetic acid by BK and AK, respectively, while pyruvate and methylmalonyl-CoA are catalyzed into oxaloacetate and propionyl CoA, respectively, by OAATC; these materials are then responsible for supplying the carbon flux from the central carbon metabolism to propionic acid. The succinic acid and propionyl CoA can also be catalyzed into succinyl-CoA and propionic acid by CoA-T. As shown in Table S6 (Supplementary Information), the key enzymes involved in the biological hydrolysis of PAM showed the highest activity at an initial fermentation pH of 9.0, which was consistent with the results observed from the biological hydrolysis of PAM.

**Figure 7:**
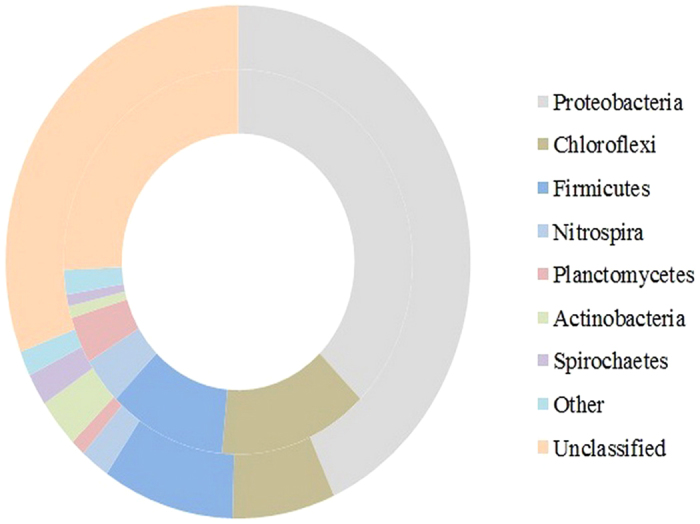
Phylum-level distributions of the *Bacterial* populations in the fermentation reactors with an initial pH of 9.0 (outer) and an uncontrolled pH (inner).

**Figure 8:**
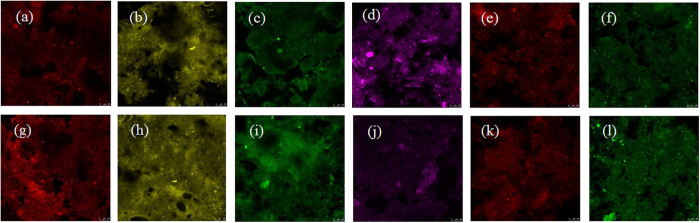
Fluorescent in situ hybridization (FISH) analysis of the key functional *Bacterial* and *Archaea* groups in the fermentation reactors with an uncontrolled pH (a–f) and an initial pH of 9.0 (g–l). An EUB mixed probe labeled with Cy-3 and FAM dyes (red in panels a and g, and yellow in panels b and h) was used to stain the domain *Bacteria*. The classes *Alphaproteobacteria* and *Betaproteobacteria* were hybridized with the probes ALF-labeled VIC (green in panels c and i) and BET42A-labeled NED (purple, in panels d and j). The ARC915 probe labeled with Cy-3 (red in panels e and k) was used to stain the domain *Archaea*. The genus of *Methanosaetaceae* was hybridized with the probe MX825-labeled FAM (green in panels f and l).

To understand the underlying mechanisms of digestion performance and the biological hydrolysis of PAM under the optimized conditions of a pH equal to 9.0, 200 mg COD/L of PAM and a fermentation time of 17 d, the key functional *Bacteria* and *Achaean* populations in the fermentation reactors with an initial pH of 9.0 and with an uncontrolled pH were determined. Figure S4 (Supplementary Information) shows the neighbor-joining phylogenetic trees of the *Bacteria* in the reactors with an initial pH of 9.0 and with an uncontrolled pH. Based on the phylum-level distributions of the *Bacteria* in the reactors with an initial pH of 9.0 and with an uncontrolled pH (Fig. 7), *Proteobacteria, Chloroflexi*, and *Firmicutes* in the reactor with an uncontrolled pH accounted for 37.6%, 13.9%, and 10.9% of the total *Bacterial* sequences present, respectively, whereas those in the reactor with an initial pH of 9.0 were determined to be 43.2%, 7.1%, and 9.2%, respectively. Additionally, a FISH analysis showed that the abundances of *Alphaproteobacteria* and *Betaproteobacteria* in the reactor with an initial pH of 9.0 accounted for 23% and 21%, respectively, which were higher than the corresponding data (e.g., 16% and 27%) in the reactor with an uncontrolled pH (Fig. 8). *Proteobacteria*, the most frequently encountered microbial group, are represented predominantly by members of *Alphaproteobacteria* and *Betaproteobacteria*, which were demonstrated to be important for the biological hydrolysis of PAM, and setting the initial pH to 9.0 increased the relative abundance of *Alphaproteobacteria*. These results indicated that setting the initial pH to 9.0 increased the relative abundance of *Proteobacteria*, which plays an important role in the biological hydrolysis of PAM. The neighbor-joining phylogenetic trees of *Archaea* present in the reactors with an initial pH of 9.0 and with an uncontrolled pH are shown in Figure S5. *Methanosaeta*, the obligate acetoclastic methanogenic *Archaea*, was found to be the most dominant *Archaea* in the reactors with an initial pH of 9.0, accounting for 70.3% of the total *Archaea* present, and in the reactors with an uncontrolled pH, accounting for 58.2% of the total *Archaea* present. Further investigation using FISH technology showed that the anaerobic reactor with an initial pH of 9.0 had significantly higher percentages of total active *Archaea* and *Methanosaetaceae* than that with an uncontrolled pH (Fig. 8). Because of the higher utilization of VFAs and other organic matter and because methane production was primarily caused by *Methanosaeta*^29^, more active *Methanosaeta* in the reactor with an initial pH of 9.0 might improve the biological hydrolysis of PAM.

The above studies introduced a new strategy to significantly enhance the anaerobic hydrolysis of PAM (i.e., amide hydrolysis) to avoid the generation of the intermediate product AM by utilizing an initial pH of 9, 200 mg COD/L of PAM and a fermentation time of 17 d. With the appropriate anaerobic fermentation conditions, the biological hydrolysis of PAM reached 86.64% using the RSM, which was much higher than that reported previously. Additionally, the mechanisms for the optimized parameters that significantly improved the biological hydrolysis of PAM were investigated by the synergistic effect of the main organic compounds in the sludge, the floc size distribution, and the enzyme activities. It was found that the biological hydrolysis of PAM could be enhanced significantly by co-fermentation with carbohydrates and proteins in the sludge. Pre-treating the sludge at pH 9.0 produced the largest contact area between the PAM and the microorganisms, which resulted in the highest key enzyme activities. Further study indicated that setting the initial pH to 9.0 increased the relative abundance of *Proteobacteria*, which plays an important role in the biological hydrolysis of PAM and improved the percentages of the total amounts of active *Archaea* and *Methanosaetaceae*.

## Methods

### Sludge and PAM

The WAS, which was concentrated by settling at 4 °C for 24 h, was collected from the secondary sedimentation tank of a municipal WWTP in Shanghai, China. The main characteristics of the concentrated sludge were as follows: pH 6.8 ± 0.2, a total amount of suspended solids (TSS) of 13890 ± 690 mg/L, an amount of volatile suspended solids (VSS) equal to 9560 ± 260 mg/L, a total amount of carbohydrates equal to 1051 ± 51 mg COD/L, and a total amount of proteins equal to 5715 ± 83 mg COD/L. Commercially produced PAM (CAS No. 9003-05-8; linear formula (C_3_H_5_NO)_n_) was purchased from Sigma Aldrich (St. Louis, MO).

### Optimized Biological Hydrolysis of PAM via Response Surface Methodology

During the anaerobic fermentation process, RSM was used to estimate the effect of certain independent variables, including the pH (*X*_*1*_), the amount of PAM (*X*_*2*_) and the fermentation time (*X*_*3*_). A five-level, three-variable central composite design (CCD) was used to guide the experiments; this model was composed of six axial points, which were coded as ± 1.68, eight factorial points, which were coded as ± 1, and six replications of center points, which were coded as ± 0. The coded independent variables used in the RSM design are shown in Table 2. The data were statistically analyzed by an ANOVA analysis using Design Expert Software (version 8.05, Stat-Ease, Inc., USA). *P* values of less than 0.05 were considered statistically significant^28^. For each test, a certain amount of PAM and 900 mL of sludge were mixed in an anaerobic reactor with a 2.0-L working volume. The pH of each reactor was adjusted to a given value by adding 5 M Ca(OH)_2_ or 4 M HCl. Each reactor was mechanically stirred at 120 rpm and maintained at 35 ± 1 °C. After a certain period of fermentation time, the mixture was sonicated at 360 W/m^2^ for 1 min with an ultrasonic generator. Then, 50 mL of the ultrasound pretreatment mixture was extracted from a reciprocal shaker at 500 rpm for 30 min at 25 °C and centrifuged at 6000 g for 15 min at 4 °C. The supernatant was then prepared for further testing. The experimental data were analyzed using multiple regressions to fit a predictive polynomial quadratic model for both responses. With the regression of the model, the parameters’ interaction effects on the biological hydrolysis of PAM (i.e., amide hydrolysis) were described with surface plots, and the optimum parameters were deduced from the fitted polynomial regression equations.

### Experiment of the Synergistic Effect of the Main Organic Compounds in Sludge on the Biological Hydrolysis of PAM

Based on the characteristics of the sludge, batch experiments were conducted using starch and bovine serum albumin (BSA) as the model polysaccharide and model protein, respectively, to investigate their roles in the biological hydrolysis of PAM. Five anaerobic reactors with diameters of 100 mm and heights of 250 mm with synthetic solutions contained the following organic compounds (mg COD/L): 200 PAM (Reactor-1), 200 PAM + 1051 Starch (Reactor-2), 200 PAM + 5715 BSA (Reactor-3), 200 PAM + 1051 Starch + 5715 BSA (Reactor-4) and 200 PAM + 1051 Starch + 5715 BSA and initial pH 9.0 (Reactor-5). These amounts of organic matter were dissolved into 800 mL of synthetic wastewater in bottles containing 405 mg/L NaHCO_3_, 155 mg/L K_2_HPO_4_·3H_2_O, 50 mg/L CaCl_2_, 100 mg/L MgCl_2_·6H_2_O, 25 mg/L FeCl_2_, 10 mg/L NaCl, 5 mg/L CoCl_2_·6H_2_O, 5 mg/L MnCl_2_·4H_2_O, 2.5 mg/L AlCl_3_, 15 mg/L (NH_4_)_6_Mo_7_O_24_, 5 mg/L H_3_BO_3_, 5 mg/L NiCl_2_·6H_2_O, 5 mg/L CuCl_2_·5H_2_O, and 5 mg/L ZnCl_2_^30^. Then, 200-mL inoculums collected from the above anaerobic reactors (Optimized Biological Hydrolysis of PAM via Response Surface Methodology) were divided equally into each anaerobic reactor and purged with nitrogen for 10 min to create anaerobic conditions. All five anaerobic reactors were then placed in an air-bath shaker at 120 rpm and 35 ± 1 °C for 17 d; these are the optimum parameters described in the section “Optimized Biological Hydrolysis of PAM using the Response Surface Methodology”. The amounts of PAM, protein and carbohydrate were then assayed.

### Comparison Among the Different Fermentation pHs Affecting Floc Size Distribution

To understand the effects of different initial pHs on the biological hydrolysis of PAM during the anaerobic fermentation process, a series of batch tests were conducted at different initial pHs (e.g., from 4.0 to 11.0). A volume of 7.2 L of WAS was divided equally into nine 2.0-L anaerobic reactors with diameters of 100 mm and heights of 250 mm, and then 200 mg COD/L of PAM was added to each reactor. The initial pHs of the eight reactors were adjusted to 4.0, 5.0, 6.0, 7.0, 8.0, 9.0, 10.0 and 11.0 by adding 5 M Ca(OH)_2_ or 4 M HCl, and the ninth reactor was set as a control with no pH adjustment. The oxygen in these anaerobic reactors was removed from the headspace by purging with nitrogen gas for 5 min. All nine anaerobic reactors were then placed in an air-bath shaker at 120 rpm and 35 ± 1 °C for 17 d, which are the optimum parameters described in the section “Optimized Biological Hydrolysis of PAM using the Response Surface Methodology”.

### Semi-Continuous-Flow Experiments to Determine the Key Enzyme Activities

Nine semi-continuous-flow reactors made of Plexiglas with working volumes of 2.0 L, internal diameters of 100 mm and heights of 250 mm were used to investigate the enzyme activity related to the biological hydrolysis of PAM, VFA production and the decomposition of proteins and carbohydrates. Then, 6.4 L of WAS was divided equally into the 8 semi-continuously operated reactors, and the initial pHs of reactors 1–8 were adjusted to equal 4.0, 5.0, 6.0, 7.0, 8.0, 9.0, 10.0 and 11.0, respectively, by adding 5 M Ca(OH)_2_ or 4 M HCl; reactor 9, which also contained 800 mL of raw sludge, was used as a control with no pH adjustment. These nine reactors were each seeded with 200 mg COD/L of PAM, and the fermentation temperature and sealing operations were the same as described above. Every day, 47 mL of the fermentation mixture was withdrawn from each reactor manually, and the same amount of sludge, which had been pre-treated or not pretreated as described above, was added; the hydrolytic retention time (HRT) of these semi-continuously operated reactors was thus 17 d. After approximately three months of operation, the biological hydrolysis of PAM remained stable; then, the enzyme activities were analyzed.

### Experimental Determination of the Key Microorganisms Involved in the Biological Hydrolysis of PAM

A batch experiment was conducted to determine the key microorganisms involved in the biological hydrolysis of PAM using an anaerobic reactor with a working volume of 2.0 L, a diameter of 100 mm and a height of 250 mm. The 40-mL inoculums collected from the above anaerobic reactors (see section “Optimized Biological Hydrolysis of PAM using the Response Surface Methodology”) were added into the anaerobic reactor after the reactor was purged with nitrogen for 10 min to create anaerobic conditions. Then, the inoculums were cultured in this anaerobic reactor with synthetic wastewater containing PAM at 200 mg COD/L as the main carbon source for 30 d. The synthetic wastewater was supplemented with buffering chemicals and inorganic nutrients as described above (see the section “Experiment of the Synergistic Effect of the Main Organic Compounds in Sludge on the Biological Hydrolysis of PAM”). The reactor was sealed with a rubber stopper, mechanically stirred at 120 rpm and maintained at 35 ± 1 °C. The samples were then collected from the anaerobic reactor after different fermentation times (e.g., 1, 4, 7, 14, 19 and 30 d) to determine the key microorganisms involved in the biological hydrolysis of PAM.

### Long-Term Reactor Operation with Regard to the Microbial Communities Present

Two semi-continuous-flow reactors with working volumes of 2.0 L were operated for a microbial community study. The two reactors, one received 800 mL of sludge with 200 mg COD/L of PAM at an initial pH of 9.0; the other received 800 mL of sludge with 200 mg COD/L of PAM without pH adjustment. Every day, 47 mL of the sludge mixture was manually removed from each reactor, and the same amount of sludge was added to each reactor. The VFAs and methane production due to the biological hydrolysis of PAM were measured during the fermentation period. After approximately 3 months of operation, the biological hydrolysis of PAM was found to remain stable; then, the microbial communities present in the reactors were analyzed.

### Analytical Methods

The amidase, alcohol dehydrogenase (ADH) and acid-forming enzymes were assayed. To determine the activities of these enzymes, 25 mL of the fermentation mixture was removed from the different anaerobic fermentation reactors and then washed and resuspended in 10 mL of 100 mM sodium phosphate buffer solution. The suspension was sonicated at 20 kHz and 4 °C for 30 min to break down the *Bacterial* cells and then centrifuged at 10000 rpm and 4 °C for 30 min to remove the waste debris. The extracts were kept on ice before they were used in the enzyme activity assays. The analytical procedures used for amidase and ADH were conducted based on the methods described by Kay-Shoemake *et al.*^31^ and Kechagias *et al.*^32^, respectively, using PAM as the substrate. The assays for phosphotransacetylase (PTA) and phosphotransbutyrylase (PTB) were based on the method described by Andersch *et al.*^33^ using acetyl-CoA and butyryl-CoA as the substrates, respectively. The acetate kinase (AK) and butyrate kinase (BK) activities were analyzed using the method of Allen *et al.*^34^ with potassium acetate and sodium butyrate as the substrates, respectively. The oxaloacetate transcarboxylase (OAATC) activity was determined based on the methods of Wood *et al.*^35^ using methylmalonyl-CoA as the substrate. The CoA transferase (CoA-T) activity was assayed using the method of Schulman and Wood^36^ with succinyl-CoA as the substrate. One unit of enzyme activity was defined to be the amount of enzyme that catalyzes the conversion of 1 μmol of substrate per minute. The specific enzyme activity was defined as the unit of enzyme activity per milligram of volatile suspended solids (VSS).

To perform the pyrosequencing, the *Bacterial* primers of 341 F and 1073 R were used^37^. PCR reactions were performed in a total volume of 20 μL, containing the template DNA (0.5 μL), Taq polymerase (2 U Ex), dNTPs (0.25 mM), Taq reaction buffer (1 × Ex, TaKaRa), MgCl_2_ (1.5 mM) and primer (5 μM). The amplification program consisted of an initial denaturation step of 94 °C for 5 min, 30 cycles of denaturation at 94 °C for 30 s, annealing at 55 °C for 30 s, and extension at 72 °C for 60 s, followed by a 5-min final extension at 72 °C. The raw sequences from the pyrosequencing process were sorted based on the specific barcodes of the sludge samples using the Pipeline Initial Process tool of the Ribosomal Database Project (RDP). The adapters, barcodes, and primers in all raw sequences were trimmed^38^, and the sequences containing ambiguous nucleotides or those shorter than 350 base pairs in length were removed^39^. The remaining sequences were checked for potential chimeras using Chimera Slayer, grouped into OTUs using the 97% identity thresholds, and compared with the SILVA 108 database.

All other analyses (i.e., TSS, VSS, SCOD, TCOD, carbohydrate, protein, VFA and methane) were conducted in the same manner as those described previously^13^. The total amount of amide-N (i.e., anaerobic hydrolysis of PAM) was measured using the starch-cadmium iodide colorimetry method^40^,^41^. The measurement of the anaerobic hydrolysis of PAM was performed as follows: the amide groups of PAM were first N-brominated with bromine to form N-bromo amide, and the excess bromine was removed from the reaction using sodium formate. Then, the N-bromo amide oxidized iodide to iodine, which was finally detected spectrophotometrically as the starch-triiodide complex. The floc size distribution analysis was performed using a Malvern Mastersizer 3000 instrument (Worcestershire, UK) with a detection range of 0.01–3500 μm. Each sample was measured three times with a standard deviation from 0.1–4.5%. The analyses of the PCR-based 16 S rRNA gene clone library and the fluorescence in situ hybridization (FISH) technique are detailed in the Supplementary Information.

### Statistical Analysis

All tests were performed in triplicate, and the significance of the results was tested by ANOVA analyses. *P* < 0.05 was considered to be statistically significant.

## Additional Information

**How to cite this article**: Dai, X. *et al.* Waste-Activated Sludge Fermentation for Polyacrylamide Biodegradation Improved by Anaerobic Hydrolysis and Key Microorganisms Involved in Biological Polyacrylamide Removal. *Sci. Rep.* **5**, 11675; doi: 10.1038/srep11675 (2015).

## Supplementary Material

Supplementary Information

## Figures and Tables

**Table 1:** Experiments with the five-level, three-variable central composite design.

**Coded levels**				**Response,** ***R***			
**Run No.**	***X*_*1*_− pH**	***X*_*2*_− PAM (mg/L)**	***X*_*3*_− time (d)**	**Maximal biological hydrolysis PAM under alkaline condition (%)**^a^		**Maximal biological hydrolysis PAM under acidic condition (%)**^b^	
				**observed**	**predicted**	**observed**	**predicted**
1	−1	1	1	69.61	74.99	82.31	84.28
2	0	0	0	91.95	86.95	80.80	76.67
3	0	1.68	0	77.32	70.34	79.36	72.98
4	−1	1	−1	31.12	37.17	42.15	46.50
5	0	0	0	89.29	86.95	79.13	76.67
6	−1.68	0	0	61.13	52.79	72.35	69.14
7	0	0	−1.68	31.68	27.49	32.41	22.25
8	−1	−1	−1	45.17	48.20	39.05	44.20
9	0	0	0	82.91	86.95	71.04	76.67
10	1	−1	−1	50.69	52.99	40.21	47.43
11	0	−1.68	0	89.72	85.84	78.25	71.63
12	1.68	0	0	63.30	60.78	80.62	70.83
13	0	0	0	89.73	86.95	72.56	76.67
14	−1	−1	1	75.65	81.44	79.79	81.16
15	1	−1	1	85.47	87.10	78.92	83.76
16	0	0	1.68	94.63	87.97	87.41	84.57
17	0	0	0	84.24	86.95	81.52	76.67
18	1	1	−1	39.12	41.01	38.09	45.91
19	1	1	1	75.06	79.70	79.01	83.06
20	0	0	0	81.69	86.95	72.72	76.67

^a^It was calculated as the biological hydrolysis removal of PAM accounting for the total PAM amount under alkaline condition.

^b^It was calculated as the biological hydrolysis removal of PAM accounting for the total PAM amount under acidic condition.

**Table 2:** Experimental range and coded levels of the independent variables.

**Coded levels**	**Variable ranges**			
	***X*_*1*_, pH (acidic)**	***X*_*1*_, pH (alkaline)**	***X*_*2*_, PAM (mg/L)**	***X*_*3*_, time (d)**
−1.68	1.64	5.64	31.82	1.86
−1	3.00	7.00	100	8.00
0	5.00	9.00	200	17.00
1	7.00	11.00	300	26.00
1.68	8.36	12.36	368.18	32.14
