# Supplementary material for: Waste-Activated Sludge Fermentation for Polyacrylamide Biodegradation Improved by Anaerobic Hydrolysis and Key Microorganisms Involved in Biological Polyacrylamide Removal
Source: Sci Rep. 2015 Jul 6;5:11675. doi: 10.1038/srep11675 (PMC4491850; doi:10.1038/srep11675)
Supplement: Supplementary Information [file srep11675-s1.doc]

**Waste-Activated Sludge Fermentation for Polyacrylamide Biodegradation Improved by Anaerobic Hydrolysis and Key Microorganisms Involved in Biological**

**Polyacrylamide Removal**

Xiaohu Dai^1^, Fan Luo ^2^, Dong Zhang*^1^, Lingling Dai^1^, Yinguang Chen^1^, Bin Dong*^1^

*(1 State Key Laboratory of Pollution Control and Resources Reuse, School of Environmental Science and Engineering, Tongji University, 1239 Siping Road, Shanghai 200092, China; 2 Guangzhou Municipal Engineering Design & Research Institute, 348 Huanshi East Road, Guangzhou, 510060)*

*Corresponding author

Dong Zhang, Phone: 86-21-55126332 Fax: 86-21-65983760 E-mail: 2008zhangdong@tongji.edu.cn

Bin Dong, Phone: 86-21-65983868 Fax: 86-21-65986313 E-mail: tj_dongbin@163.com

**Supplementary Information:** 14 pages, 9 tables, 5 figures

**Materials and Methods**

**PCR-based 16S rRNA gene clone library.** Before the analysis of microbial community with the biological hydrolysis of PAM, the sludge mixture was centrifuged at 10000 rpm for 5 min, and the total genomic DNA was extracted according to Purkhold et al. (*1*). The centrifuged precipitate (0.5 g) was washed three times with STET buffer (Sucrose 8%, Triton X-100 5%, EDTA 50 mM, Tris 50 mM, pH 8) and then re-suspended in 360 μL STET buffers. The mixture was incubated at 37℃ for 10 min, after adding with 40 μL lysozyme (50 mg/mL). A total 20 μL SDS (sodium dodecyl sulfate, 10%) and 2 uL proteinase K (20 mg/mL) were added, and then the mixture was incubated at 37°C for 60 min. A total 50 μL NaCl (sodium chloride, 5 M) and 50 μL CTAB (cetyl trimethyl ammonium bromide, 10%) were added and then incubated at 65°C for 10 min. After that, 0.5 mL Tris-saturated phenols, 0.5 mL phenol-chloroform-isoamyl alcohols (25:24:1), and 0.5 mL chloroform-isoamyl alcohols (24:1) was used, respectively, to extract the nucleic acids, which were then precipitated by incubation with 0.1 volume of NaAc (sodium acetate, 3 M, pH5.2) and 2 volumes of ethanol for 1 h at room temperature and subsequently centrifuged at 13000 rpm for 10 min. The pellets were washed with 500 μL ethanol (70%), dried at room temperature, and finally re-suspended in 50 μL elution buffer (10 mMTris, pH 8.5). The extracted DNA was checked by 1% agarose electrophoresis (ethidium bromide was using as the staining dye). The nearly complete 16S rRNA gene fragments were amplified using the primers 27F (5′-AGAGTTTGATCCTGGCTCAG-3′) and 1492R (5′-GGTTACCTTGTTACGACTT-3′) for *Bacterial* sequences and 21F (5′-TTCCGGTTGATC CYGCCGGA-3′) and 1041R (5′-GGCCATGCACCWCCTCTC-3′) for archaeal sequences (*2*). PCR amplification was carried out in a total volume of 25 μL containing Taq reaction buffer (1×Ex), template DNA (10 ng), MgCl_2_ (3.0 mM), Taq polymerase (2U Ex), primers (0.5 μM, TaKaRa) and dNTPs (0.2 mM). The amplification program consisted of an initial denaturation step of 94°C for 5 min, 30 cycles of denaturation at 94°C for 30 s, annealing at 55°C for 30 s, and extension at 72°C for 60 s, followed by a 5 min final extension at 72°C. The amplified DNA was ligated into the pMD19-T vector (TaKaRa), and transformed into *Escherichia coli* DH5R cells (TaKaRa) with ampicillin selection and blue/white screening. Multiple alignments were generated using the ClustalX 2.0, and then the phylogenetic tree was constructed with MEGA 4.0 using the Jukes-Cantor model for the neighbor-joining algorithm.

**FISH technique with the 16S rRNA-targeted oligonucleotide probes.** According to the literature the following 16S rRNA-targeted oligonucleotide probes were used in this study: EUB338mix for *Bacteria*, ALF for *Alphaproteabacteria* (*3*), BET42A for *Betaproteobacteria* (*4*), ARC915 for *Archaea* (methanogens) (*5*), MX825 for *Methanosaetaceae* (*6*). The information about these probes is listed in Table S7 (Supplementary Information), and the FISH procedures were conducted the same as our previous publication (*7*). After hybridization at 46°C for 10 h, the specimens were stained with 4’, 6’-diamidino-2-phenylindole (DAPI) (1 mg/mL). Then, the sections hybridized with the probes were observed with a confocal laser scanning microscope (CLSM, Leica TCS, SP2 AOBS). Ten at-random fields were analyzed to determine the average numbers of cells in the samples.

**Table S1. Reports of PAM utilization as a nitrogen or a carbon source.**

| **Biodegradation pathway** | **Condition** | **Object** | **Literatures** |
| --- | --- | --- | --- |
| Nitrogen source | Anaerobic | Soil | Kay-Shoemake *et al.*, 1998, (*8*) |
| Nitrogen source/  Partly carbon source | Aerobic | Oilfield wastewater | Bao *et al.*, 2010, (*9*) |
| Carbon source | Aerobic | Oilfield wastewater | Wen *et al.*, 2011, (*10*) |
| Nitrogen source | Aerobic | PAM gel | Holliman *et al.*, 2005, (*11*) |
| Nitrogen source | Anaerobic | Sewage sludge/Tailings | Haveroen *et al.*, 2005, (*12*) |
| Carbon source | UV+ Anaerobic/Aerobic | Sewage sludge | El-Mamouni *et al.*, 2002, (*13*) |
| Carbon source | Aerobic | Soil/ Sewage sludge | Nakamiya *et al.*, 1995, (14) |
| Recalcitrance to biodegradation | Anaerobic | Sewage sludge | Chu *et al.*, 2003, (*15*) |
| Recalcitrance to biodegradation | O_3_+ Aerobic | River bed mud | Suzuki *et al.*, 1978, (*16*) |

**Table S2. ANOVA results of the response surface quadratic model of the biological hydrolysis of PAM.**

|  | Model | | Std. Dev. | *R^2^* | Adj.*R^2^* | Pred.*R^2^* | Adeq Precision | C.V. % |
| --- | --- | --- | --- | --- | --- | --- | --- | --- |
|  | *F*-value | *P*-value |
| *R_alkaline_* | 19.81 | <0.0001 | 6.57 | 0.9469 | 0.8991 | 0.6645 | 13.010 | 9.33 |
| *R_acidi_c* | 10.29 | 0.0006 | 7.86 | 0.9025 | 0.8148 | 0.3696 | 11.218 | 11.49 |

**Table S3. Effect of the fermentation pH on the sludge floc median diameter and the biological hydrolysis of PAM.**

| Initial pH | 4.0 | 5.0 | 6.0 | 7.0 | 8.0 | 9.0 | 10.0 | 11.0 |
| --- | --- | --- | --- | --- | --- | --- | --- | --- |
| Median particle size (D_50_) ^a^ | 253±8 | 183±5 | 270±9 | 294±8 | 134±4 | 109±4 | 122±3 | 133±4 |
| Hydrolysis PAM removal ^b^ | 75.8±2.4 | 77.1±3.2 | 74.6±2.0 | 72.1±2.1 | 79.6±2.9 | 86.8±3.4 | 85.1±2.4 | 80.5±3.3 |
| ^a^ The unit is um. The data are the averages and their standard deviations in duplicate tests.  ^b^ The unit is %. The data are the averages and their standard deviations in duplicate tests. | | | | | | | | |

**Table S4. Sequence diversity and library coverage estimates^a^.**

| Fermentation time (d) | 1 | 4 | 7 | 14 | 19 | 30 |
| --- | --- | --- | --- | --- | --- | --- |
| Number of valid sequence | 3695 | 3658 | 3879 | 3627 | 4313 | 4214 |
| Operational taxonomic units (OTUs) | 684 | 764 | 740 | 762 | 957 | 722 |
| Shannon index diversity | 4.96 | 5.23 | 5.14 | 5.22 | 5.30 | 4.40 |
| Simpson index diversity | 0.022 | 0.015 | 0.019 | 0.018 | 0.020 | 0.075 |
| sufficient coverage (%) | 88.7 | 87.5 | 89.2 | 87.4 | 86.3 | 89.4 |
| ^a^ It was defined by the 97% identity threshold (i.e., 3 % dissimilarity level).. | | | | | | |

**Table S5. Pearson correlation between the biological hydrolysis of PAM and the microbial community.**

|  | | | | | | |
| --- | --- | --- | --- | --- | --- | --- |
| Phylum | *Bacteroidetes* | *Firmicutes* | *Proteobacteria* | *Spirochaetes* | *Synergistetes* | *Thermotogae* |
| PAM removal rate | -0.124 | -0.060 | 0.860* | 0.059 | -0.229 | -0.379 |
| *Correlation is significant at the 0.05 level. | | | | | | |

**Table S6. Effect of the fermentation pH on the key enzyme activities involved in biological hydrolysis of PAM ^a^**

| pH value | AM ^b^ | ADH | AK | BK | PTA | PTB | OAATC | CoA-T |
| --- | --- | --- | --- | --- | --- | --- | --- | --- |
| 4.0 | 1.6157±0.0507 | 0.161±0.0071 | 0.7345±0.0192 | 0.0509±0.0015 | 0.0057±0.0002 | 0.0145±0.0006 | 0.4729±0.0116 | 0.1892±0.0068 |
| 5.0 | 1.6577±0.0528 | 0.1723±0.0066 | 0.8630±0.0232 | 0.0505±0.0012 | 0.0068±0.0003 | 0.0154±0.0005 | 0.513±0.0156 | 0.2684±0.0093 |
| 6.0 | 2.1029±0.0803 | 0.1457±0.0053 | 0.8447±0.0199 | 0.057±0.0018 | 0.0072±0.0003 | 0.0166±0.0007 | 0.5349±0.0167 | 0.2891±0.0107 |
| 7.0 | 2.4221±0.0811 | 0.1982±0.0079 | 0.8814±0.0337 | 0.0649±0.0021 | 0.0118±0.0005 | 0.0174±0.0007 | 0.5698±0.0205 | 0.2995±0.0083 |
| 8.0 | 2.1533±0.0780 | 0.2134±0.0087 | 0.9181±0.0358 | 0.0719±0.0025 | 0.0168±0.0006 | 0.0179±0.0008 | 0.5795±0.0196 | 0.3104±0.0112 |
| 9.0 | 4.2193±0.1542 | 0.3813±0.0142 | 0.9916±0.0378 | 0.075±0.0027 | 0.0278±0.0009 | 0.0194±0.0008 | 0.6074±0.0237 | 0.3592±0.0126 |
| 10.0 | 3.4553±0.1330 | 0.3728±0.0134 | 1.1017±0.0395 | 0.0786±0.0019 | 0.0236±0.0008 | 0.0208±0.0009 | 0.6157±0.0187 | 0.3439±0.0116 |
| 11.0 | 2.1365±0.0860 | 0.3533±0.0125 | 0.9893±0.0294 | 0.0752±0.0021 | 0.0231±0.0007 | 0.0203±0.0008 | 0.6023±0.0167 | 0.3095±0.0096 |
| ^a^ Data are the averages and their standard deviations in three different measurements. The unit of enzyme activity is U / mg VSS.  ^b^ The unit of AM activity is g NH_4_^+^-N_released_/g VSS. | | | | | | | | |

**Table S7. Oligonucleotide probes used in this study.**

| Probe | Specificity | Sequence (5’-3’) of probe | Formamide content (%) |
| --- | --- | --- | --- |
| EUB338 | *Bacteria* | GCTGCCTCCCGTAGGAGT | 20 |
| EUB338-II |  | GCAGCCACCCGTAGGTGT | 20 |
| EUB338-III |  | GCTGCCACCCGTAGGTGT | 20 |
| ARC915 | *Archaea* | GTGCTCCCCCGCCAATTCCT | 35 |
| MX825 | *Methanosaetaceae* | TCGCACCGTGGCCGACACCTAGC | 20 |
| ALF | *α proteabacteria* | GAAATCCCACATGCTTT | 35 |
| Bet42A | *β proteobacteria* | GCCTTCCCACTTCGTTT | 35 |

**Table S8. The coefficient of the quadratic models for the responses of the biological hydrolysis of PAM under alkaline or acidic conditions via ANOVA.**

|  | Biological hydrolysis PAM  under alkaline condition | | | |  | Biological hydrolysis PAM  under acidic condition | | | |
| --- | --- | --- | --- | --- | --- | --- | --- | --- | --- |
| Factor | Coefficient | Std. Error | *F*-value | *P*-value |  | Coefficient | Std. Error | *F*-value | *P*-value |
| Intercept | 86.95 | 2.68 |  |  |  | 76.67 | 3.20 |  |  |
| *X_1_,* pH | 2.38 | 1.78 | 1.78 | 0.2114 |  | 0.50 | 2.13 | 0.055 | 0.8185 |
| *X_2_,* PAM | -4.61 | 1.78 | 6.71 | 0.0269 |  | 0.40 | 2.13 | 0.035 | 0.8547 |
| *X_3_,* Time | 17.98 | 1.78 | 102.16 | <0.0001 |  | 18.53 | 2.13 | 75.95 | <0.0001 |
| *X_1_X_2_* | -0.24 | 2.32 | 0.010 | 0.9210 |  | -0.96 | 2.78 | 0.12 | 0.7378 |
| *X_1_X_3_* | 0.22 | 2.32 | 0.009 | 0.9269 |  | -0.16 | 2.78 | 0.003 | 0.9556 |
| *X_2_X_3_* | 1.15 | 2.32 | 0.24 | 0.6326 |  | 0.20 | 2.78 | 0.005 | 0.9430 |
| *X_1_^2^* | -10.66 | 1.73 | 37.91 | 0.0001 |  | -2.36 | 2.07 | 1.30 | 0.2802 |
| *X_2_^2^* | 3.13 | 1.73 | 3.27 | 0.1009 |  | -1.54 | 2.07 | 0.56 | 0.4732 |
| *X_3_^2^* | -10.33 | 1.73 | 35.58 | 0.0001 |  | -8.22 | 2.07 | 15.79 | 0.0026 |

**Table S9. The statistical analysis results of the synergistic effect of the main organic compounds in sludge on the biological hydrolysis of PAM^a^.**

|  | Items | F_observed_ | F_significance_ | P _(0.05)_ |
| --- | --- | --- | --- | --- |
| PAM + Starch | PAM removal | 108.14 | 7.71 | 0.0005 |
| PAM + BSA | PAM removal | 127.71 | 7.71 | 0.0003 |
| PAM + Starch + BSA | PAM removal | 175.27 | 7.71 | 0.0002 |
| PAM + Starch + BSA + pH9 | PAM removal | 477.67 | 7.71 | 0.00003 |
| *^a^* The fermentation time was 17 d. | | | | |

**
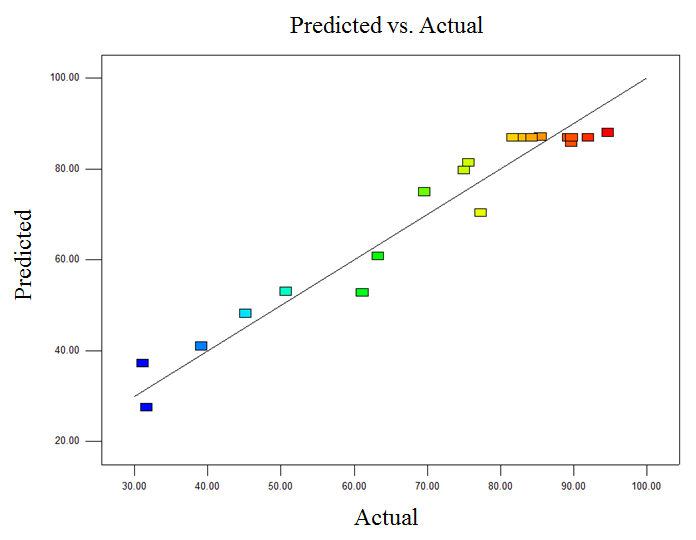

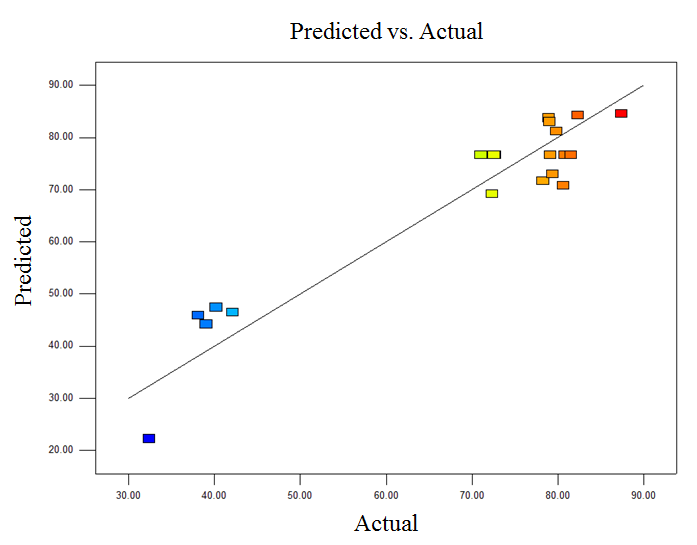
**

(a) (b)

**Figure S1 Predicted versus actual plots of the biological hydrolysis of PAM in the alkaline (a) and acidic conditions (b).**

**
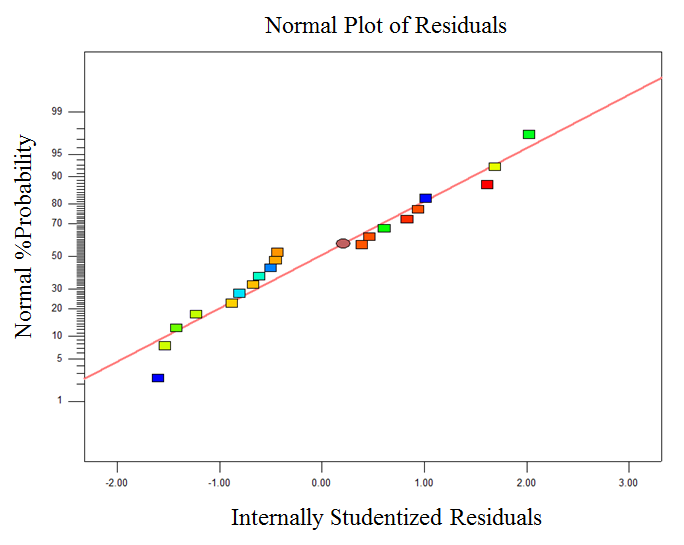

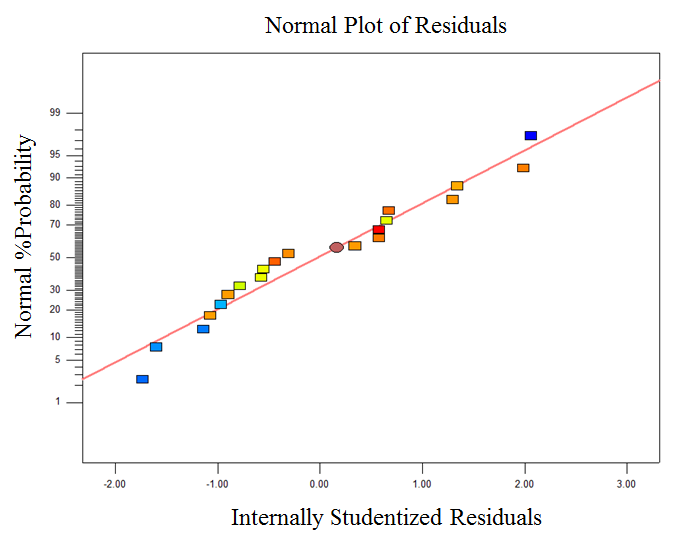
**

(a) (b)

**Figure S2 Normal probability of the residual for** **biological hydrolysis of PAM in the alkaline (a) and acidic conditions (b).**

**
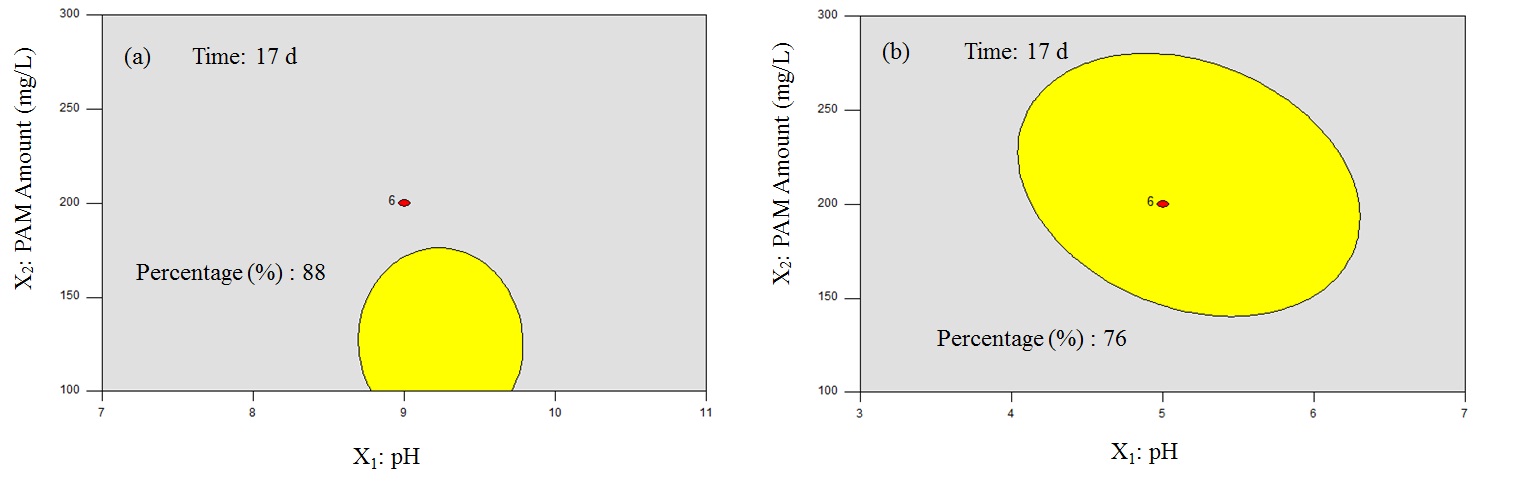
**

**Figure S3 Overlay plots for biological hydrolysis of PAM by setting the amount of PAM and pH as variable factors after 17 day cultivation in alkaline (a) and acidic conditions (b).**

*Acidovorax* AB076844

*Diaphorobacter* AB166774

*Curvibacter* AB286395

*Comamonas* EU864453

*Dechloromonas* AF331976

*Thauera* AB066262

*Aquimonas* EU809367

*Dokdonella* JF808836

*Steroidobacter* AM934731

*Hyphomicrobium* AB487448

*Sphingomonas* EU937899

*Rhizomicrobium* EU104123

*Rhodobacter* AF234761

*Brevundimonas* GQ199733

*Bauldia* AY921890

*Beijerinckia* AJ289986

*Methylocystis* AJ458472

*Syntrophorhabdus* EU399674

*Bellilinea* AB445103

*Longilinea* AJ249113

*Caldilinea* AB630576

*Nitrospira* JN679171

*Treponema* AF050549

*Iamia* GQ500882

*Propionicicella* EU266885

*Bacillus* DQ212969

*Enterococcus* EU034531

*Gracilibacter* AJ488067

*Anaerovorax* AY57063

*Acetoanaerobium* AJ488068

*Clostridium XI* DQ325984

*Planctomyces* FJ542870

100

100

70

56

100

98

100

95

99

99

96

96

62

49

64

92

55

99

98

87

76

85

73

77

46

49

37

32

32

0.05

*Delta-proteobacteria* 1.02%

*Anaerolineae* 4.08%

*Caldilineae* 3.06%

*Nitrospira* 2.04%

*Spirochaetes* 2.04%

*Actinobacteria* 3.06%

*Bacilli* 5.10%

*Clostridia* 4.08%

*Planctomycetacia* 1.02%

(a) pH 9.0

*Comamonas* EU864453

*Curvibacter* AB286395

*Acidovorax* AB076844

*Piscinibacter* EU834815

*Georgfuchsia* AF204243

*Thauera* AB066262

*Azospira* AY823971

*Dechloromonas* AF331976

*Azonexus* KC551713

*Aquimonas* EU809367

*Steroidobacter* AM934731

*Paracoccus* AB025188

*Rhodobacter* AF234761

*Filomicrobium* DQ395499

*Hyphomicrobium* AB487448

*Bradyrhizobium* AY624133

*Methylocystis* AJ458472

*Anaerofilum* GU559778

*Clostridium sensu stricto* DQ248269

*Bacillus* DQ212969

*Syntrophorhabdus* EU399674

*Iamia* GQ500882

*Nitrospira* JN679171

*Caldilinea* AB630576

*Bellilinea* AB445103

*Longilinea* AJ249113

*Treponema* AF050549

*Blastopirellula* FJ203616

*Planctomyces* FJ542870

100

100

96

93

95

98

53

88

96

72

32

42

80

100

81

87

63

79

82

95

81

50

73

41

63

25

0.05

*Beta-proteobacteria* 22.77%

*Gamma-proteobacteria* 3.96%

*Alpha-proteabacteria* 9.90%

*Clostridia* 4.95%

*Bacilli* 5.94%

*Delta-proteobacteria* 0.99%

*Actinobacteria* 0.99%

*Nitrospira* 3.96%

*Caldilineae* 8.91%

*Anaerolineae* 4.95%

*Spirochaetes* 0.99%

*Planctomycetes* 3.96%

(b) uncontrolled pH

**Figure S4 Neighbor-joining phylogenetic tree of the *Bacteria* present in the reactors with an initial pH of 9.0 and with an uncontrolled pH.** The scale bar represents 0.05 substitutions per nucleotide position.

*Methanolinea*

*Methanosphaerula*

*Methanospirillum*

*Methanoregula*

*Methanosaeta*

*Methanosarcina*

*Methanobacterium*

*Methanosphaera*

100

99

99

59

59

0.05

EU155919 1.85%

EU910630 1.85%

CP000254 18.52%

EU591661 70.37%

EU888806 3.70%

AY695843 1.85%

AF424765 1.85%

EU155919 0

(a) pH 9.0

*Methanolinea*

*Methanosphaerula*

*Methanospirillum*

*Methanoregula*

*Methanosaeta*

*Methanosarcina*

*Methanobacterium*

*Methanosphaera*

100

99

99

59

59

0.05

EU155919 1.81%

EU910630 5.45%

CP000254 14.55%

EU591661 58.18%

EU888806 1.81%

AY695843 5.45%

AF424765 9.09%

EU155919 3.64%

(b) uncontrolled pH

**Figure S5 Neighbor-joining phylogenetic tree of *Archaea* present in the reactors with an initial pH of 9.0 and with an uncontrolled pH.** The scale bar represents 0.05 substitutions per nucleotide position.

**References**

1. Purkhold, U. *et al.* Phylogeny of all recognized species of ammonia oxidizers based on comparative

16S rRNA and amoA sequence analysis: Implications for molecular diversity surveys. *Appl. Environ. Microbiol.* **66**, 5368–5382 (2000).

2. Lane, D. 16S/23S rRNA Sequencing. *In Nucleic Acid Techniques in Bacterial Systematics*;

Stackebrandt, E., Goodfellow, M., Eds., 115–147, (John Wiley & Sons: Chichester, U. K., 1991).

3. Glöckner, F. O., Fuchs, B. M. & Amann, R. Bacterioplankton compositions of lakes and oceans: a

first comparison based on fluorescence *in situ* hybridization. *Appl. Environ. Microbiol.* **65**,

3721–3726 (1999).

4. Manz, W., Amann, R., Ludwig, W., Wagner, M. & Schleifer, K. H. Phylogenetic

oligodeoxynucleotide probes for the major subclasses of proteobacteria-problems and solutions.

*Syst. Appl. Microbiol.* **15**, 593–600 (1992).

5. Stahl, D. A., Flesher, B., Mansﬁeld, H. R. & Montgomery, L. Use of phylogenetically based

hybridization probes for studies of ruminal microbial ecology. *Appl. Environ. Microbiol.*

**54**, 1079–1084 (1988).

6. Raskin, L., Stromley, J. M., Rittmann, B. E. & Stahl, D. A. Group-speciﬁc 16S rRNA hybridization

probes to describe natural communities of methanogens. *Appl. Environ. Microbiol.* **60**, 1232–1240 (1994).

7. Zhang, D., Chen, Y., Zhao, Y. & Ye, Z. A new process for efficiently producing methane from waste

activated sludge: alkaline pretreatment of sludge followed by treatment of fermentation liquid in an

EGSB reactor. *Environ. Sci. Technol.* **45**, 803–808 (2011).

8. Kay-Shoemake, J. L., Watwood, M. E. & Lentz, R. D. Polyacrylamide as an organic nitrogen

source for soil microorganisms with potential impact on inorganic soil nitrogen in agricultural

soil. *Soil Biol. Biochem.* **30**, 1045–1052 (1998).

9. Bao, M., Chen, Q. & Li, Y. Biodegradation of partially hydrolyzed polyacrylamide by bacteria

isolated from production water after polymer flooding in an oil field. *J. Hazard. Mater*. **184**,

105–110 (2010).

10. Wen, Q., Chen, Z. & Zhao, Y. Performance and microbial characteristics of bioaugmentation systems for polyacrylamide degradation. *J. Polym. Environ*. **19**, 125–132 (2011).

11. Holliman, P. J., Clark, J. A. & Williamson, J. C. Model and field studies of the degradation of

cross-linked polyacrylamide gels used during the revegetation of slate waste. *Sci. Total. Environ.*

**336**, 13–24 (2005).

12. Haveroen, M. E., MacKinnon, M. D. & Fedorak, P. M. Polyacrylamide added as a nitrogen source

stimulates methanogenesis in consortia from various wastewaters. *Water Res*. **39**, 3333–3341

(2005).

13. El-Mamouni, R., Frigon, J. C. & Hawari, J. Combining photolysis and bioprocesses for mineralization of high molecular weight polyacrylamides. *Biodegradation* **13**, 221–227 (2002).

14. Nakamiya, K. & Kinoshita, S. Isolation of polyacrylamide-degrading bacteria. *J. Fermentation*

*Bioeng*. **80**, 418–420 (1995).

15. Chu, C. *et al.* Anaerobic digestion of polyelectrolyte flocculated waste activated sludge. *Chemosphere* **53**, 757–764 (2003).

16. Suzuki, J., Hukushima, K. & Suzuki, S. Effect of ozone treatment upon biodegradability of water-soluble polymers. *Environ. Sci. Technol.* **72**, 1180–1183 (1978).
